# The Impact of Violence and Abuse on Mental Health of Women – Current Data

**DOI:** 10.1192/j.eurpsy.2022.130

**Published:** 2022-09-01

**Authors:** M. Schouler-Ocak, E.J. Brandl

**Affiliations:** Charité – Universitätsmedizin Berlin, Psychiatric University Clinic At St. Hedwig Hospital, Berlin, Germany

**Keywords:** violence against women, mental health, current data, role of mental health professionals

## Abstract

Violence against women is widely recognised as a violation of human rights and a public health problem. The most common forms of violence against women are domestic abuse and sexual violence, and victimisation is associated with an increased risk of mental disorders. It is reported that a three times increase in the likelihood of depressive disorders, a four times increase in the likelihood of anxiety disorders, and a seven times increase in the likelihood of post-traumatic disorder (PTSD) for women who have experienced domestic violence and abuse. Significant associations between intimate partner violence and symptoms of psychosis, substance misuse, and eating disorders have also been reported. Furthermore, systematic reviews of predominantly cross-sectional studies report consistent relationships between being a victim of domestic violence and abuse and having mental disorders across the diagnostic spectrum for men and women, but since women are more likely to be victims, the population attributable fractions are higher for women. In this presentation, the focus will also be on clinical guidance on the role of mental health professionals in identifying violence against women and responding appropriately, poor identification persists and can lead to non-engagement with services and poor response to treatment. After a literature review, we will present and discuss current data from parental consultation and a survey on violence during the Covid-19 pandemic in Berlin.

**Disclosure:**

No significant relationships.

